# *In vitro* blood cell viability profiling of polymers used in molecular assembly

**DOI:** 10.1038/s41598-017-10169-5

**Published:** 2017-08-25

**Authors:** Hyejoong Jeong, Jangsun Hwang, Hwankyu Lee, Paula T. Hammond, Jonghoon Choi, Jinkee Hong

**Affiliations:** 1 School of Chemical Engineering & Materials Science, Chung-Ang University, 84 Heukseok-ro, Dongjak-gu, Seoul, 06974 Republic of Korea; 2 School of Integrative Engineering, Chung-Ang University, 84 Heukseok-ro, Dongjak-gu, Seoul, 06974 Republic of Korea; 3 Department of Chemical Engineering, Dankook University, 152 Jukjeon-ro, Suji-gu, Yongin-si, Gyeonggi-do, 16889 Republic of Korea; 4 School of Chemical Engineering, Koch Institute for Integrative Cancer Research, Massachusetts Institute of Technology, Cambridge, MA 02139 USA

## Abstract

Biocompatible polymers have been extensively applied to molecular assembly techniques on a micro- and nanoscale to miniaturize functional devices for biomedical uses. However, cytotoxic assessments of developed devices are prone to partially focus on non-specific cells or cells associated with the specific applications. Thereby, since toxicity is dependent on the type of cells and protocols, we do not fully understand the relative toxicities of polymers. Additionally, we need to ensure the blood cell biocompatibility of developed devices prior to that of targeted cells because most of the devices contact the blood before reaching the targeted regions. Motivated by this issue, we focused on screening cytotoxicity of polymers widely used for the layer-by-layer assembly technique using human blood cells. Cytotoxicity at the early stage was investigated on twenty types of polymers (positively charged, negatively charged, or neutral) and ten combination forms via hemolysis, cell viability, and AnnexinV-FITC/PI staining assays. We determined their effects on the cell membrane depending on their surface chemistry by molecular dynamics simulations. Furthermore, the toxicity of LbL-assembled nanofilms was assessed by measuring cell viability. Based on this report, researchers can produce nanofilms that are better suited for drug delivery and biomedical applications by reducing the possible cytotoxicity.

## Introduction

Biomaterials are either derived from nature or synthesized using polymers, ceramics, metals, and composite materials. Specifically, polymers have been extensively applied to controlled release systems since 1976, when Langer *et al*. developed a cornea assay that continually released macromolecules to inhibit angiogenesis^1^. In recent decades, polymers have been manipulated by the layer-by-layer (LbL) assembly technique to create controlled release systems with miniaturized biomedical devices on a simple and tiny scale. The LbL assembly is a promising molecular assembly technique to fabricate multilayer thin films based on alternating deposition of oppositely charged polymers^2^. This process has introduced a new way to fabricate multicomponent and ultrathin films on curved surfaces of nano- and microparticles. Thus, nanoparticles for use as drug delivery carriers have been commonly prepared using the LbL technique with a variety of polymers, active biomolecules, and functional materials^3–5^. There have been many studies on drug delivery, but *in vitro* and *in vivo* toxicity assessments for each developed carrier are required. Most reports have assessed cytotoxicity using only target cells or non-specific cells from animals. This approach cannot represent the overall toxicity for humans because of the differences between many of the cells used in these studies and human cells.

To overcome these limitations, Choksakulnimitr *et al*. (1995) evaluated the *in vitro* cytotoxicity of several macromolecules in macrophages, brain microvessel endothelial cells (BMECs), and hepatocytes from mice or rats. They assessed lactate dehydrogenase (LDH)-release to determine the cytotoxic effects of macromolecules based on their electric charges^6^. Kissel *et al*. (2003) also assessed the *in vitro* cytotoxicity of various biomaterials in L929 mouse fibroblasts. They found that the cytotoxic effect and mechanism of each polymer were due to various factors (electric charge, molecular weight, and chemical structure) using different assays^7^. Cytotoxicity information is helpful for drug delivery research to predict and determine the cytotoxic effect of newly developed compounds. For this reason, these reports have been cited thousands of times. However, they were unable to evaluate a broad range of polymers, and polymer toxicity has not been extensively studied with human cells.

**Figure 1:**
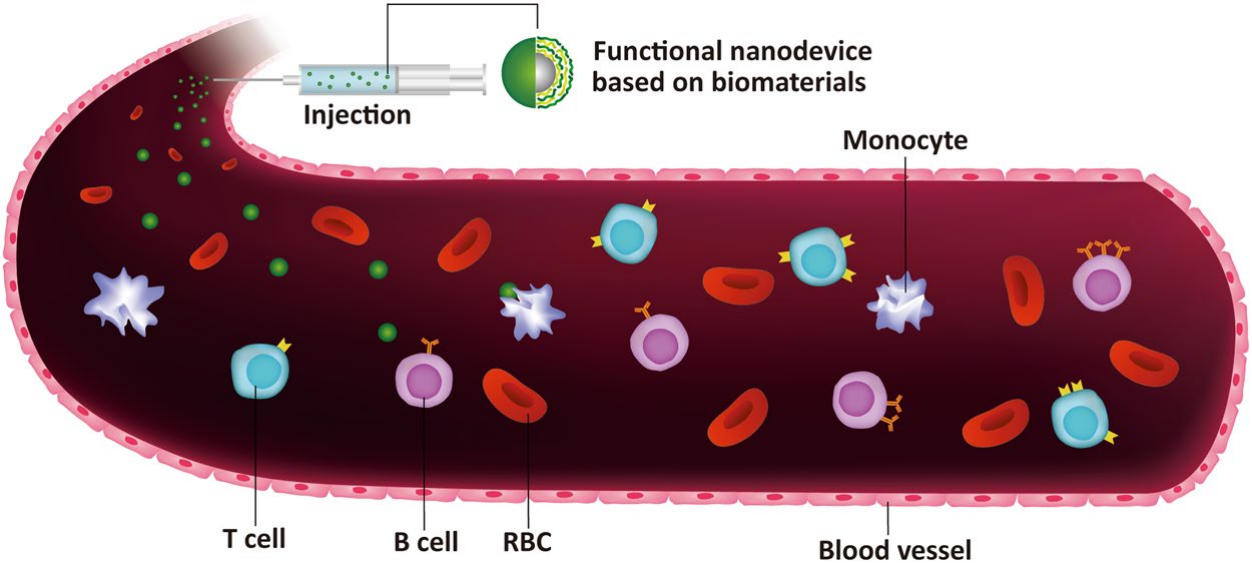
Scheme of the first stage of an *in vivo* study of functional nanodevices prepared from biomaterials.

Motivated by the lack of studies on polymers, we investigated the cytotoxic effects of polymers frequently used in the LbL assembly technique. We used red blood cells (RBCs) and a group of immunological cells, peripheral blood mononuclear cells (PBMCs) in an attempt to overcome the limitations of previous reports that were restricted to animal and normal cells. In an *in vivo* study, the first point to consider is the blood. When devices used for drug delivery, targeting, imaging, and diagnosis are injected *in vivo*, they initially contact the blood, which is composed of hemocytes and immune cells (Fig. 1). If a device shows toxicity to blood cells, it naturally would not be viable for biomedical use. Thus, the toxic effects of nanodevices should be assessed with regard to the blood system prior to uncharacterized or target cells.

Here, we demonstrate the blood compatibility of twenty types of polymers via *in vitro* toxicity profiling using RBCs and PBMCs derived from humans. RBCs are the most common cell type in blood, comprising approximately 45% by volume of blood, and PBMCs are a group of immune cells consisting of lymphocytes, including T cells, B cells, NK cells, and monocytes. We implemented three types of assays: a hemolysis assay using RBCs, cell viability and AnnexinV-FITC/propidium iodide (PI) staining assays using PBMCs.

A hemolysis assay is an indispensable initial step in evaluating the blood compatibility of polymers to identify severe acute toxic reactions in RBCs *in vivo* ^8^. Hemolysis refers to the disruption of RBCs, and the assay detects the leaking of intracellular contents including hemoglobin into the plasma. Hemoglobin is an essential protein in RBCs and plays a significant role in transporting oxygen from the respiratory organs to the rest of the cells and tissues. However, hemoglobin released through hemolysis becomes a vasoactive and redox active protein that has toxic effects on vascular, myocardial, renal, and central nervous system tissues, inducing anemia, jaundice, and other pathological conditions^9^. Many studies have reported that *in vitro* hemolysis assays have good correlations with *in vivo* toxicity by the hemolytic effect^10^. Thus, this report is a preliminary investigation of the *in vivo* toxicity of polymers using the results of *in vitro* hemolysis assays.

Taking full advantage of uncharacterized PBMCs, we conducted a cell viability assay as a preliminary study to assess biocompatibility and immunotoxicity of polymers. Here, we suggest several reasons for choosing uncharacterized PBMCs for this study. First, PBMCs include lymphocytes (T cells, B cells, and NK cells) and monocytes, which have nuclei, and do not include macrophages, erythrocytes, and platelets. PBMCs are cultured while floating and do not require any substrates for anchoring, and we could investigate the early stage of polymer toxicity. Second, death of PBMCs could be considered as a surrogate of cytokine release and immunotoxicity. This is because cytokines associated with inflammation are released from the cells when the PBMCs enter the apoptotic phase^8^. In fact, PBMCs have been widely used in many fields, such as immunology, infectious diseases, hematological malignancies, vaccine development, transplant immunology, and high-throughput screening for drug candidates. Therefore, the cytotoxicity of PBMCs could represent the potential of immune reaction and immunotoxicity of polymers, and we can predict the immune effects of drug delivery systems prepared by polymers during preclinical safety evaluations using *in vitro* assessments^11^. Third, there is not much research regarding the cytotoxic effects of LbL polymer structures in terms of immunotoxicity using uncharacterized PBMCs. Since our goal was to observe the response of untouched PBMCs near *ex vivo* conditions, directly separated from blood, it was important that we used uncharacterized PBMCs for this study.

The AnnexinV-FITC/PI staining assay is used to determine the state of cell death. This assay discriminates between intact cells (FITC^−^/PI^−^), apoptotic cells (FITC^+^/PI^−^), and necrotic cells (FITC^+^/PI^+^)^12^. In the early stages of apoptosis, phosphatidylserine normally located on the inner membrane is exposed on the outer membrane of the cell surface; AnnexinV-FITC then binds to the phosphatidylserine with high affinity. After the cell membrane has disintegrated, PI is able to reach the nucleus and bind with DNA. Therefore, AnnexinV-FITC directly indicates cells in the early stage of apoptosis, and PI indicates cells in the late apoptotic stage that progresses to necrosis^12, 13^.

Following the hypothesis of the effects of injecting drug delivery systems in the blood, we implemented our assays in the order of hemolysis, cell viability, and AnnexinV-FITC/PI staining assays. When external material enters the intravascular system of the human body, it certainly encounters blood cells, including RBCs and PBMCs (the rest of blood without RBCs composed of neutrophils, monocytes, eosinophils, lymphocytes, plasma cells, basophils, and platelets). The polymers used in this study were divided into three groups based on their electric charge: polycations, polyanions, and neutral polymers (Table 1). Additionally, we assessed the toxicity of the polymers as combinations and films. Combinations were composed of polycations and polyanions, commonly used LbL assembly technique (Table S1). Based on these findings, we believe that our report provides a guideline for the toxicity and safety of biomaterials used by researchers in the field of LbL assembly, as well as in all fields that use biomaterials.

**Table 1:** Physicochemical properties of the polymers used.

Material	Charge/monomer ratio^d^	Monomer Mw	Mw	pKa
PDAC^a^	0.006194	161.45	100,000~200,000	—
BPEI^a^	0.216666	60	~25,000	9.36
PLL^a^	0.015625	128	70,000~150,000	9.4~10.5
GO(+)^a^	0.001887	4,239.48	—	PI 10.7
COL^a^	—	—	—	PI 8.2
PAAM^a^	—	71	5,000,000	—
LPEI^a^	0.013333	75	250,000	5
PBAE^a^	0.011905	252	~10,000	—
PAH^a^	0.010701	93.45	~15,000	8.5
CHI^a^	0.006173	162	Low	6.2
TA^b^	0.005878	1,701.20	1700	2.5
FUCO^b^	0.002041	490	20,000	2.0
PSS^b^	0.004854	206	~75,000	—
DEX^b^	0.005917	338	Mr 2,000,000	2.0
HA^b^	0.002639	379	—	2.9
PAA^b^	0.013889	72	1,800	4.5
GO(−)^b^	0.002839	4227.48	—	4.2
HEP^b^	0.006745	593	—	3
PEG^c^	—	62	200	—
PVA^c^	—	44	89,000~98,000	—

^*^Polycation^a^, polyanion^b^, and neutral polymer^c^. *The charge/monomer ratio^d^ is calculated as the number of charges/monomer Mw.

## Results and Discussion

### Polymer toxicity profiling analysis

**Figure 2:**
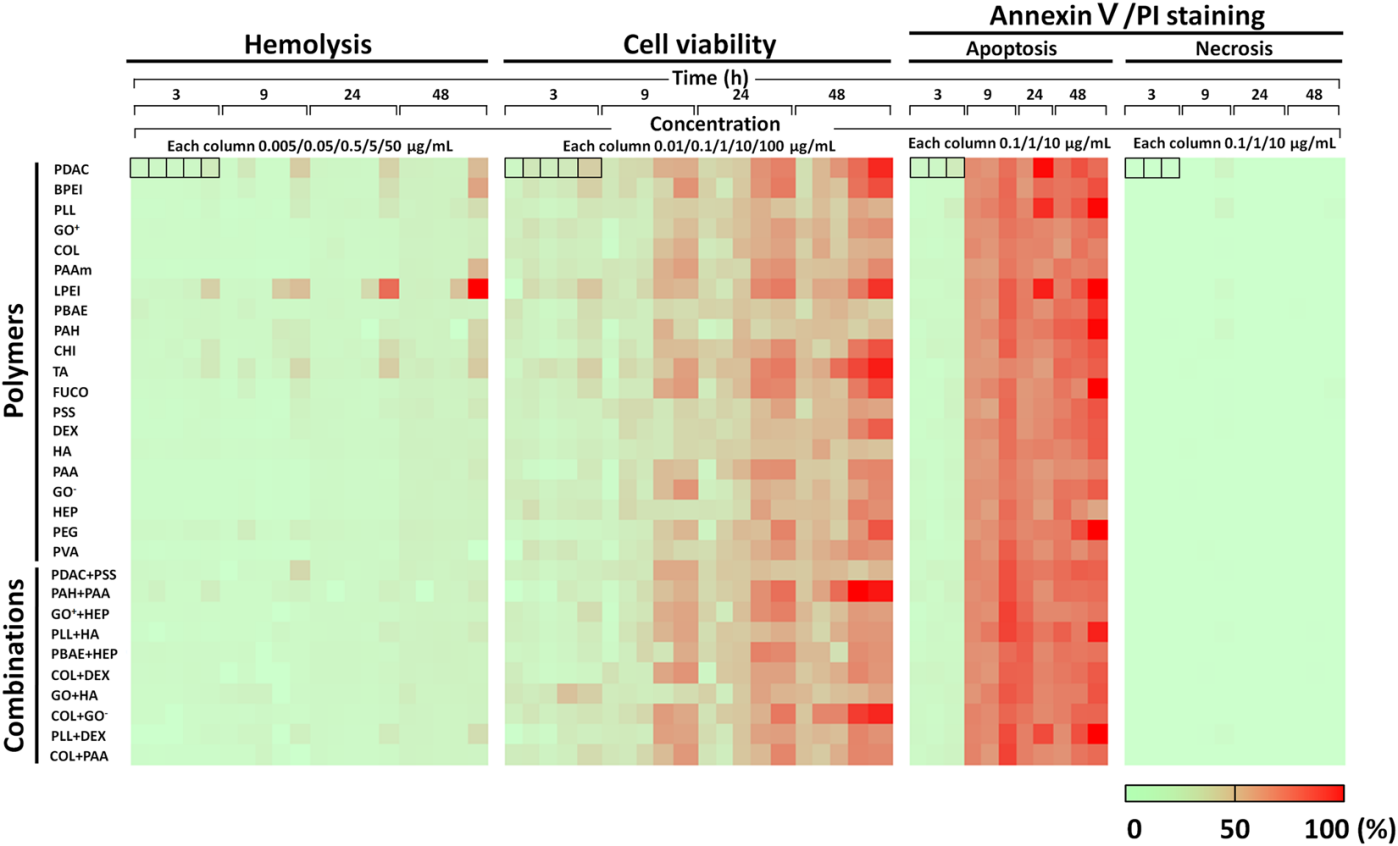
Heat map indicating the cytotoxic effects of whole materials according to concentration and time. The colors indicate the relative cytotoxicity levels (percentages of hemolysis, cell death, and apoptotic or necrotic cells) for each polymer and combination under 64 different conditions in the rows (all polymers and combinations of three/five doses × four culture times × three assays).

As shown in Fig. 2, we assessed each selected polymer and combination under different conditions as follows: (i) Three or five concentrations of each material were used over a range of 0.005 to 50 μg/mL for hemolysis, 0.01 to 100 μg/mL for cell viability, and 0.1 to 10 μg/mL for AnnexinV-FITC/PI staining. We used high concentrations to account for bioaccumulation. In the AnnexinV-FITC/PI staining assay, only a practically effective range of concentrations (0.1 to 10 μg/mL) was examined. (ii) The four culture times (3, 9, 24, 48 h) covered approximately 1 min of blood circulation time corresponding to an average blood circulation rate of 5.25 L/min and considering 12 h of clearance time in the human body^14^. A long culture time would reflect an unexpected situation. (iii) Three assays were used to measure hemolysis, cell viability, and AnnexinV-FITC/PI staining (detects apoptotic or necrotic cells).

The overall results in the heat map are arranged by relative cytotoxicity and indicated by colors. The columns present each assay in order of cell culture times and different concentrations. The toxicity levels of each polymer can be easily compared using the colors of the overall heat map. In the hemolysis assay, most polymers showed no cytotoxicity, and in most cases in the cell viability assay, cell death increased with polymer concentration and culture time. In the AnnexinV-FITC/PI staining assay, apoptosis occurred in a time-dependent manner without a necrotic reaction. Details on the individual toxicity of the materials will follow.

### Cytotoxicity of polymers

**Figure 3:**
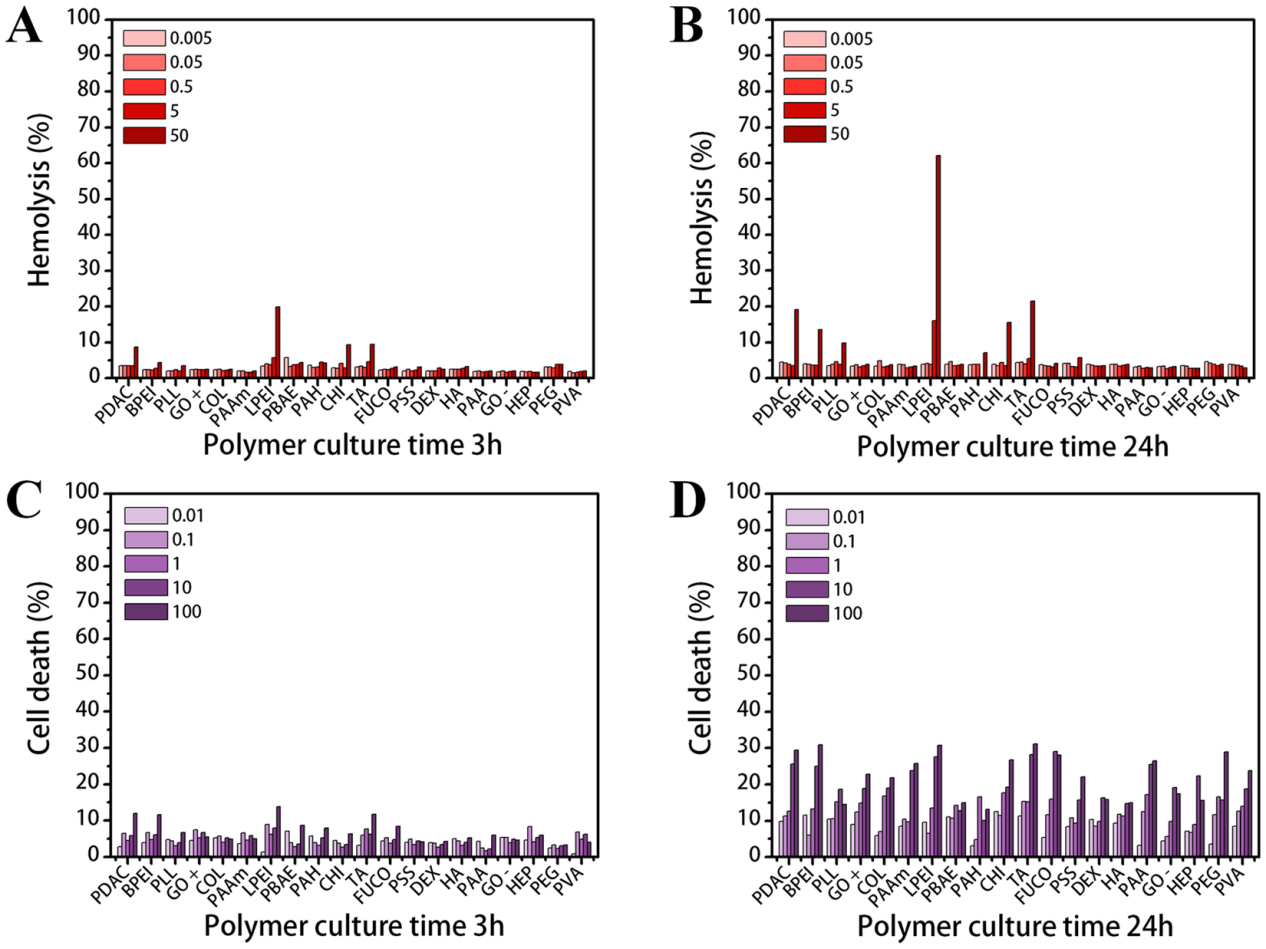
Cytotoxic effects of polymers on RBCs and PBMCs. Hemolysis ratios of different polymer concentrations ranging from 0.005 to 50 µg/mL after (**A**) 3 h and (**B**) 24 h. Cell death ratios after exposure to five polymer concentrations from 0.01 to 100 µg/mL for (**C**) 3 h and (**D**) 24 h. The negative control is PEG.

To examine the cytotoxic effects of each polymer, the results of two assays for 3 and 24 h are presented in Fig. 3. The results for 9 and 48 h are shown in Figure S1. To predict the toxic effects on an *in vivo* system, an early cytotoxic response (within 3 h) and cytotoxicity after 24 h are essential when considering blood circulation and clearance time. Although doses over 5 µg/mL would be too high to be used practically, we assessed this range as well to account for the accumulation of the polymers in certain tissues or organs. The overall results show that the polymers generally have a harmful effect on cells depending on their concentration and exposure time. In Fig. 3A, cytotoxicity is indicated at over 10% hemolysis^7^, and only LPEI at its highest dose showed mild toxicity. After 24 h (Fig. 3B), toxicity was indicated at the higher concentrations of several polymers in the order of LPEI (62.1%) > TA (21.5%) > PDAC (19.2%) > CHI (15.6%) > BPEI (13.6%) at 50 µg/mL. After 48 h (Figure S1B), the toxic effects of the above polymers continuously increased; additionally, the higher concentrations of PAAM and PAH showed toxicity. However, most of the toxic effects were limited to higher concentrations, longer exposure times, and cationic polymers. Based on these results, we can infer that RBCs are prone to be lysed when a large number of polycations attack their membrane.

Using PBMCs, we evaluated the cytotoxic effects of polymers on cells and the related cell death states. Cell viability was assessed by the dye exclusion method using trypan blue, which is a dye that stains only dead cells. After 3 h, no cytotoxic effects were observed in the PBMCs (Fig. 3C and D). After 24 h, cell death increased in a concentration-dependent manner. For a more distinct analysis, the amount of cell death is presented in a normalized graph with comparison to PEG as a negative control (Figure S2A–D). At the early culture times, most polymers induced more cell death than the control. Over 24 h, with some exceptions, the cytotoxicity levels were almost in the same range. Based on this result, we surmised that the rate of action depends on the electric charge. According to the higher cell death ratio for polycations (left side) shown in Figure S2A and C, polycations acted on the cells very quickly, inducing cell death within 9 hours. Cell death occurred slowly in the presence of polyanions and PEG. Nevertheless, the final cytotoxic effects were similar due to the strong influence of higher concentrations and longer exposure times. We concluded that the rate of the cytotoxic action among the groups mostly depended on the charge of the polymers, as the cell membrane is negatively charged.

In the AnnexinV/PI staining assay results shown in Fig. 3, because few cells reacted with PI, we did not include necrotic cell data. Thus, no polymer induced severe toxicity to achieve continuous membrane disruption and necrosis.

**Figure 4:**
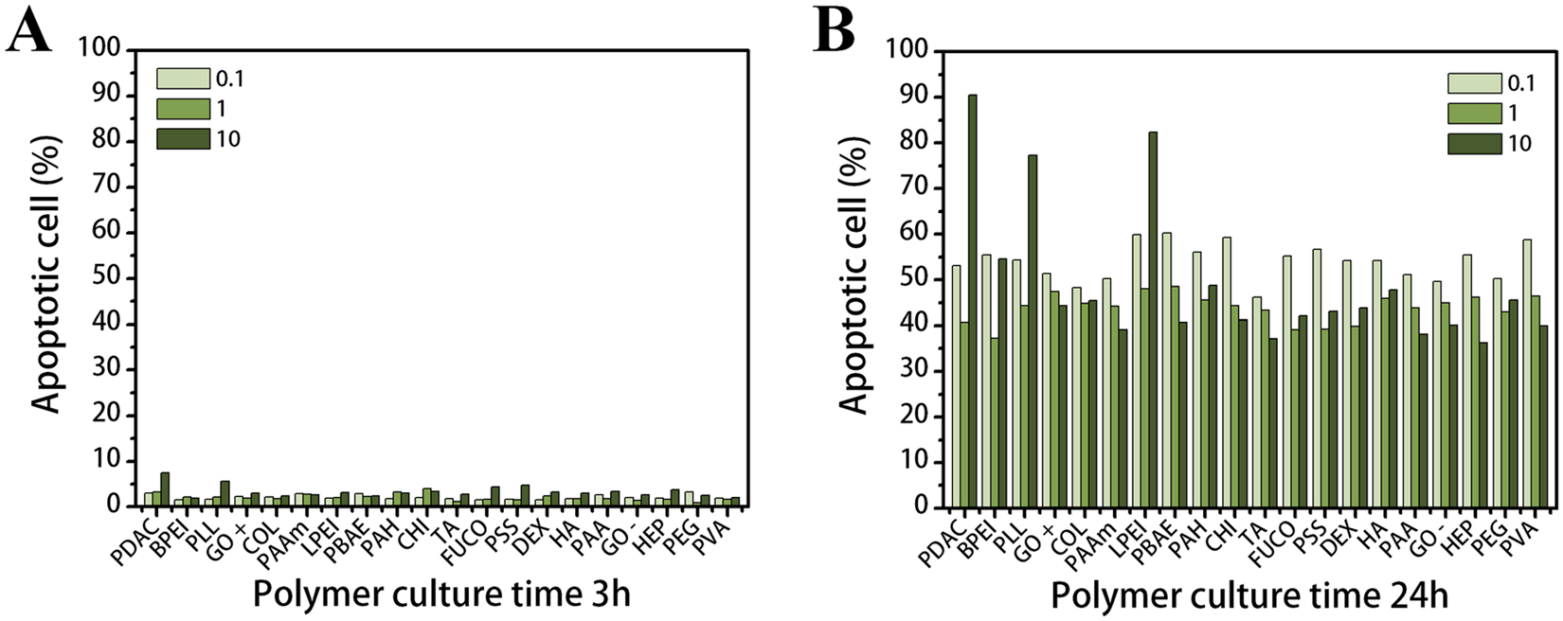
Cytotoxic effects of polymers on PBMCs. Apoptotic cell ratios of three polymer concentrations from 0.1 to 10 µg/mL after (**A**) 3 h and (**B**) 24 h. Negative control is PEG.

After 9 h (Fig. 4A), a large proportion of cells exposed to PDAC (90.5%), LPEI (82.4%), and PLL (77.3%) at 10 µg/mL were altered to apoptotic forms compared to PEG (45.6%). This result corresponds to those from the hemolysis and cell viability assays. These results confirm that polycations directly damage the cell membrane. In Figure S1F, the ratio of apoptotic cells is over 50%; however, PEG also showed a higher ratio (92%) at 10 µg/mL. This indicates that although PEG is non-toxic and non-immunogenic, the early apoptotic cell phase could be caused by the high amounts of polymers on the cell surface.

The results shown in Figs 3 and 4 indicate that no severe cytotoxic effects are caused by polymers within effective ranges of concentration and time. However, at high concentrations (over 50 µg/mL) and particularly with increased time, several polymers, including LPEI, TA, PDAC, CHI, BPEI, and PLL, were cytotoxic against both RBCs and PBMCs. In fact, various polymer factors can induce cytotoxicity, such as high molecular weight, electric charge, degree of ionization (DOI), structure, and functional groups^7^. Our results show that most of the toxic polymers had a positive charge due to amine groups and a high molecular weight (>70,000). Polycations can easily bind to the cell surface, which is composed of negatively charged lipid bilayers, and cause damage to cells^6, 7^. Nevertheless, LPEI and CHI were not ionized at pH 7.4 in a physiological environment according to their pKa values in Table 1. Taking this into consideration, we must assume that not only electric charges but also other factors can have a harmful effect on cells.

PEIs including LPEI and BPEI showed a strong cytotoxic effect due to their large molecular mass and high charge density resulting from a large number of secondary amine groups. According to a report from Fischer *et al*., high molecular weight PEI (800 kD) induced massive necrosis within 30 minutes and low molecular weight PEI (25 kD) had low cytotoxicity^15^. PEI is well known for its cell transfection properties, but its cytotoxicity increases as its molecular weight increases. Similarly, PDAC has a large molecular weight and a high cationic charge density that both affect cells^16^. Kean *et al*. reported that the toxicity of CHI is related to the charge density of its secondary amine groups. However, low cytotoxicity can occur because a threshold level is not met when there are few contact points between a polymer and cells^17^. The toxicity of PLL results from higher molecular weight and secondary amine groups. Choksakulnimitr *et al*. found that high molecular weight (39,800 kD) PLL showed a higher toxicity than low molecular weight PLL (8000 kD)^6^. TA is a plant polyphenol known to be an antioxidant and was the only toxic polyanion in our study. Additionally, Labieniec *et al*. reported that 60 µM TA showed the highest toxicity with over 50% of cell death as well as DNA damage against B14 cells. The mechanism of TA toxicity has not been elucidated; however, we believe that the large number of hydroxyl groups in TA impart strong antioxidant and pro-oxidant properties, producing metabolites, including ellagic and gallic acids, that have a toxic effect on the cells^18, 19^.

To better understand the cytotoxic mechanisms of the polymers, we focused on LPEI and PLLs with various charges to determine the effects of chemical structure and electric charge through molecular dynamics simulations.

### Molecular dynamics simulations of PEI and PLL in lipid bilayers

**Figure 5:**
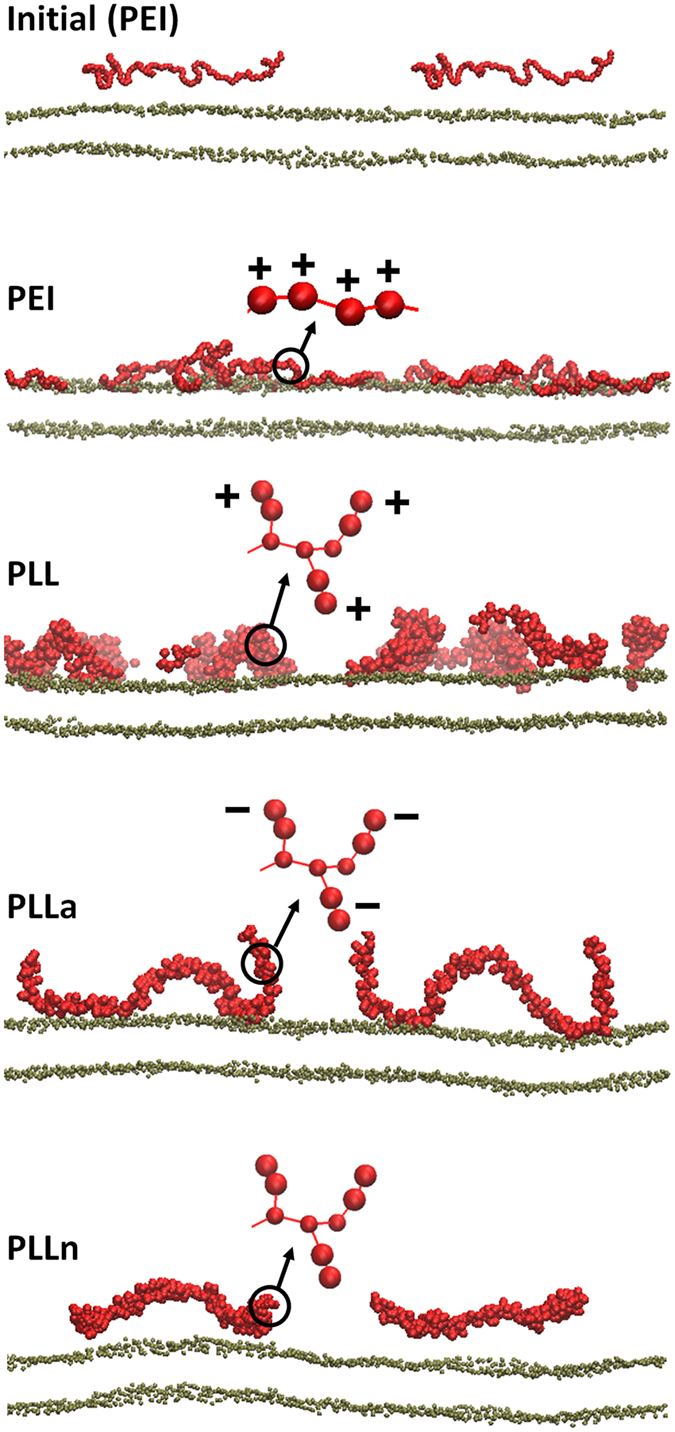
Snapshots of the side view at the beginning (0 ns; top) and the end (400 ns; rows 2–5) of the simulations. The initial configuration is shown only for PEI, but for other systems, the polymer chains are also similarly distributed above the bilayer surface. Red and brown colors represent polymers and lipid phosphates, respectively. Schematic structures of polymers are shown with charges, whereas lipid tails, water, and counter ions (Na^+^ or Cl^−^) are omitted for clarity. Note that the side views show only one cross-section of the system and cannot capture all eight polymer chains. The images were created using Visual Molecular Dynamics^25^.

**Figure 6:**
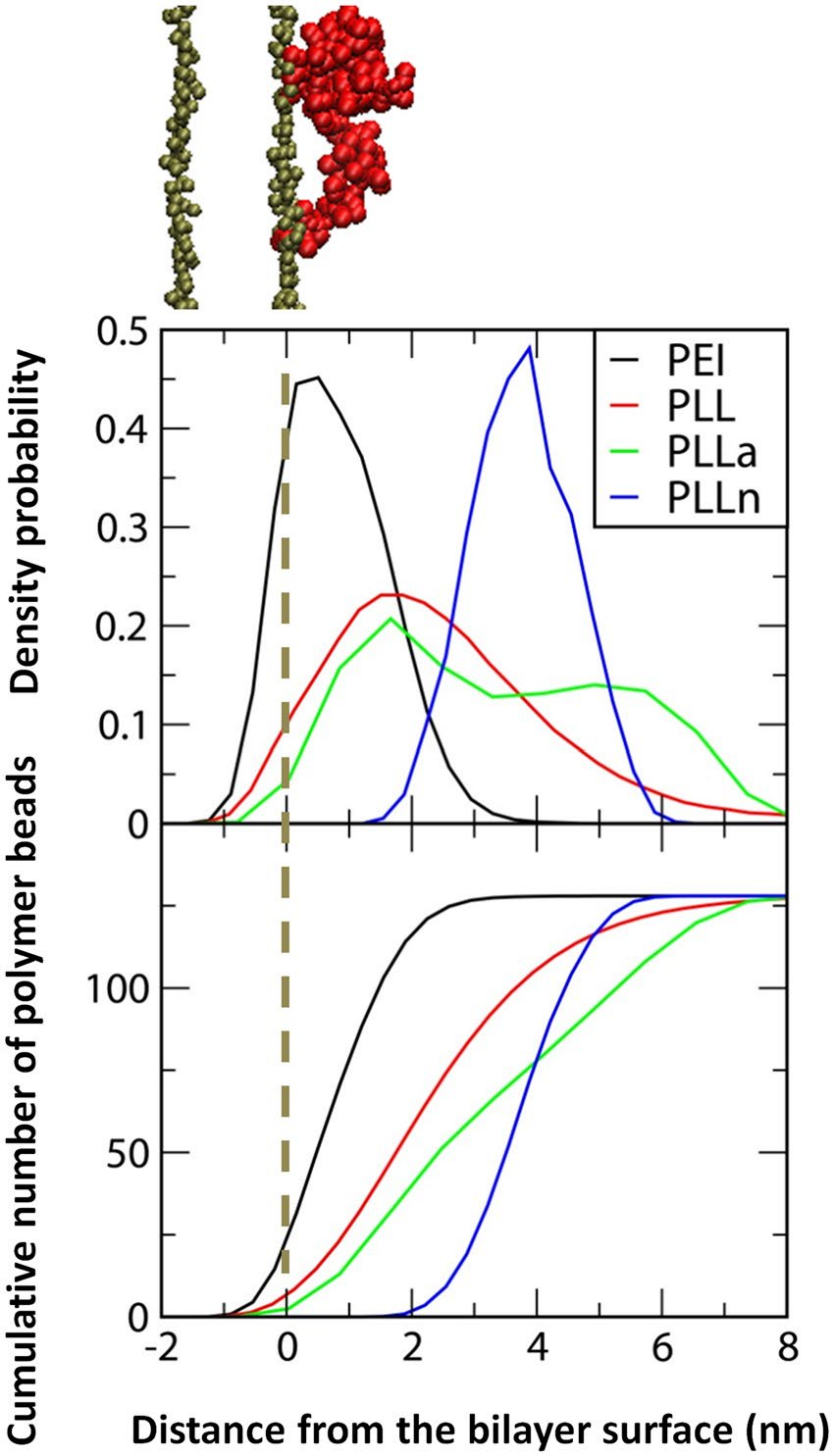
Density probabilities of polymer chains on the bilayer surface as a function of distance from the z-directional center of mass of lipid-phosphate beads in the adjacent leaflet (top) and the cumulative numbers of charged (or neutrally mutated) beads (bottom).

Linear random-coil polymers with different charges and structures were simulated in lipid bilayers with explicit water for 400 ns. Note that the simulated lipid bilayers differ from realistic cellular membranes composed of various lipid components and protein receptors. Despite this limitation, simulations of lipid bilayers have successfully reproduced experimental observations regarding the cytotoxicity of proteins and nanoparticles, and thus the dimyristoylglycerophosphocholine (DMPC) bilayer was used as a model membrane in this work^20, 21^. The simulated systems are listed in Table 2. The letters “a” and “n” following PLL indicate the polymers modified with anionic and neutral beads, respectively, to replace the cationic beads of PLL side chains. For example, “PLLn” designates the bilayer system with the neutrally mutated PLL. Figure 5 shows the initial and final snapshots of the simulations. Starting with the polymers above the bilayer surface, PEI, PLL, and PLLa bind to the bilayer surface, while PLLn does not, indicating that the polymer charge affects the interaction between polymers and bilayers. In particular, PEI and PLL bind more tightly to the bilayer than PLLa chains, indicating a stronger interaction with cationic polymers than with anionic polymers. This is expected, as anionic polymers cannot interact with anionic lipid phosphates^22^, whereas cationic polymers do and thus can insert more deeply into the bilayer^23, 24^. These configurations and the binding extent of the polymers were also confirmed by calculating density profiles. In Fig. 6, the PEI chains are mostly located in the lipid head-group region, whereas PLLn chains are positioned farthest from the head-group region, showing that the adsorption order (PEI > PLL > PLLa > PLLn) is consistent with Fig. 5. The cumulative numbers of the charged (or neutrally mutated) beads of polymers were also calculated as a function of the distance from the bilayer surface, showing that the number of PEI beads drastically increases near the bilayer surface, whereas those of other polymers do not, as shown in the density profiles. These results indicate that cationic polymers more strongly interact with lipid bilayers than do anionic polymers and that neutral polymers do not bind to the bilayer. This finding supports the above-mentioned experimental results regarding the different extents of toxicity for various polymers.

**Table 2:** List of simulations.

	Name	No. of charges per molecule		Adsorption onto the bilayer
		Cation	Anion	
Polyethylenimine	PEI	128	—	√
Poly- l-lysine (PLL)	PLL	128	—	√
Mutated PLL	PLLa	—	128	√
	PLLn	—	—	—

**Figure 7:**
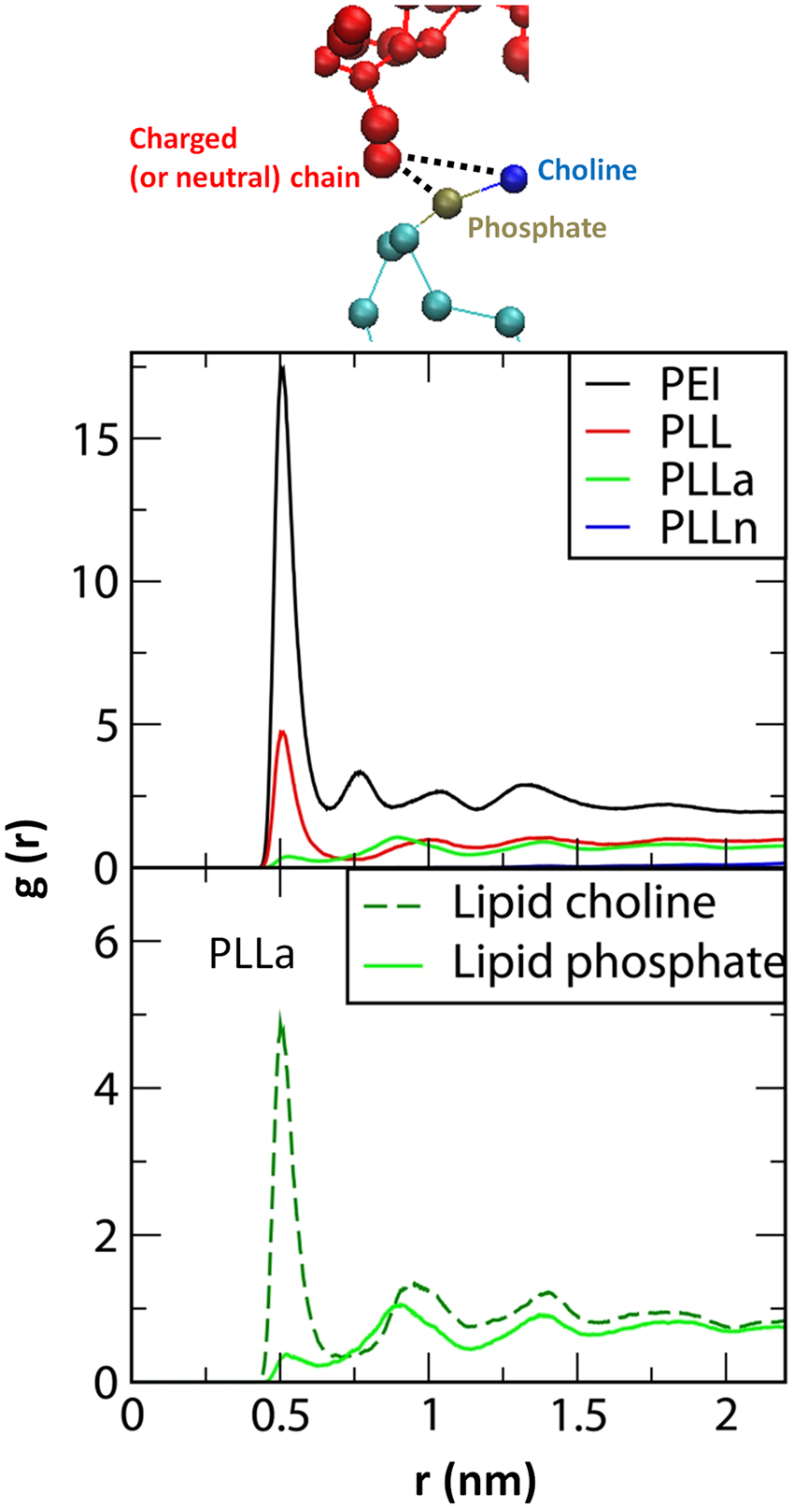
Radial distribution functions between charged (or neutrally mutated) polymer beads and lipid phosphates (top) and between the anionic beads of PLLa and lipid head groups (bottom).

Because cationic polymers have a stronger interaction with lipid bilayers, the different charge states of polymers may modulate the bilayer properties. To answer this question, the polymer-bilayer interactions were further quantified by calculating radial distribution functions (RDFs) between lipid phosphates and the charged (or neutrally mutated) beads of the polymer. Figure 7 shows the peak heights in the order of PEI > PLL > PLLa > PLLn, indicating that cationic polymers have stronger electrostatic interactions with the bilayer surface, consistent with Figs 5 and 6. Note that although PEI and PLL have the same positive charge density, the RDF peak is much higher for PEI than for PLL. This is apparently because PLL chains consist of backbones and side chains, whereas PEI chains contain only backbones, so the charged beads of PEI are more accessible to the bilayer surface than the charged beads of PLL side chains, as visualized in Fig. 5. In Fig. 7 (bottom), the RDFs between lipid head groups and PLLa side chains show that the anionic beads of PLLa tend to electrostatically interact with cationic cholines of lipids rather than with anionic phosphates, which explains why there is less adsorption of anionic polymers onto the bilayer in Fig. 6.

These simulation findings indicate that charged polymers strongly interact with lipid bilayers, whereas neutral polymers do not, illustrating the effect of polymer charge on the interaction between polymers and lipid bilayers. In particular, cationic and anionic polymers interact with lipid phosphates and cholines, respectively, leading to a stronger adsorption of cationic polymers onto the bilayer. Additionally, our simulations show that linear polymers with only charged backbones (PEI) have a stronger electrostatic interaction with the bilayer than those with backbones and charged side chains (PLL) because the charged beads of the former are sterically more accessible to the lipid head groups. This dependence on the structure and charge state of linear polymers agrees well with the above experimental results showing a higher toxicity for cationic polymers than for anionic and neutral polymers and that linear PEI is more toxic than non-linear PLL.

### Cytotoxicity of polymer combinations

**Figure 8:**
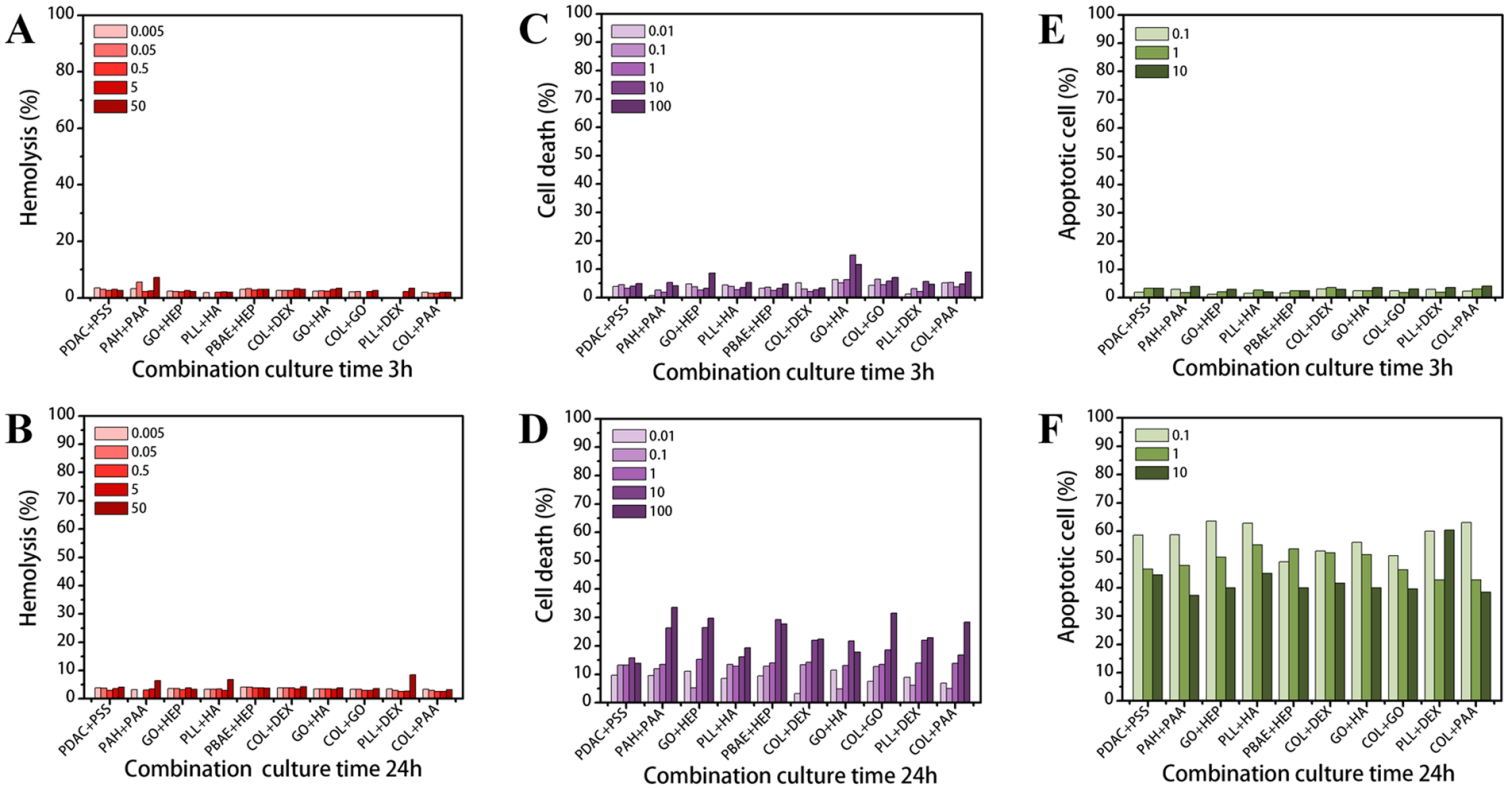
Cytotoxic effects of polymer combinations on RBC and PBMCs. Hemolysis ratios of different combination concentrations ranging from 0.005 to 50 µg/mL after (**A**) 3 h and (**B**) 24 h. Cell death ratios after exposure to five combination concentrations ranging from 0.01 to 100 µg/mL after (**C**) 3 h and (**D**) 24 h. Apoptotic cell ratios after exposure to three combination concentrations ranging from 0.1 to 10 µg/mL after (**E**) 3 h and (**F**) 24 h. Negative control is PEG.

As shown in Fig. 8, there was no severe toxicity from any combination. Additional results after 9 and 48 h are presented in Figure S3. Despite low hemolysis levels, some of the combinations, including PAH + PAA and COL + GO, showed higher cell death ratios after 48 h (Figure S3D). In addition, PLL + HA and PLL + DEX showed higher apoptotic cell levels at 10 µg/mL after 48 h (Figure S3F). Figure S4 shows the overall cytotoxicity results of the combinations normalized to the results for PEG. According to these results, we conclude that there are no toxicity tendencies among the three assays and that the combinations generally had less cytotoxicity than polymers alone. We hypothesize that the reason for this finding is that opposite charges are counterbalanced, eliminating the electric charges seen in the polymers. To confirm this hypothesis, we investigated the electric charges of combinations under physiological conditions at pH 7.4 using the zeta potential method, as shown in Table S1. In this simulation, we assumed that positively charged combinations would show a stronger cytotoxic effect on cells. However, our results did not indicate any toxic effects according to the charges. There are several possible reasons explaining why the combinations have less cytotoxicity. First, aggregated colloidal shapes could cause less physical damage to the cell surface. Second, the zeta potential was not a sufficient measurement to evaluate the counterbalanced charges of the combinations because it recognizes solutions as particle shapes. Third, the movement of individual polymers was restricted due to aggregation via electrostatic interactions.

Based on these results, we expect that LbL films fabricated using combinations will be more biocompatible than individual polymers. To verify this assumption, four types of LbL films were assessed for their effects on the viability of PBMCs.

### Cytotoxicity of LbL films

To examine the cytotoxic effect of polymers incorporated into LbL films, four types of films in different compositions were prepared at thicknesses of 30 and 100 nm. Based on the above results, the first combinations were composed of toxic polymers, including PDAC, FUCO, BPEI, and TA. The second combinations were prepared using non-toxic polymers, including PAH, PSS, COL, and HA. Thus, the toxic combinations were PDAC/FUCO and BPEI/TA, whereas the non-toxic combinations were PAH/PSS and COL/HA. To exclude the surface charge effect on cytotoxicity, every film was terminated with negatively charged polymers. In addition, we hypothesized that when the films are disintegrated in the aqueous solution, the disintegration rate varies depending on the thickness, and cytotoxic effects from degradation products may differ. Therefore, we prepared both 30-nm and 100-nm films. We predicted that apoptosis would occur by toxic combinations and thick films, provided that direct cell-surface interactions and disintegration products are the cause of cell death.

**Figure 9:**
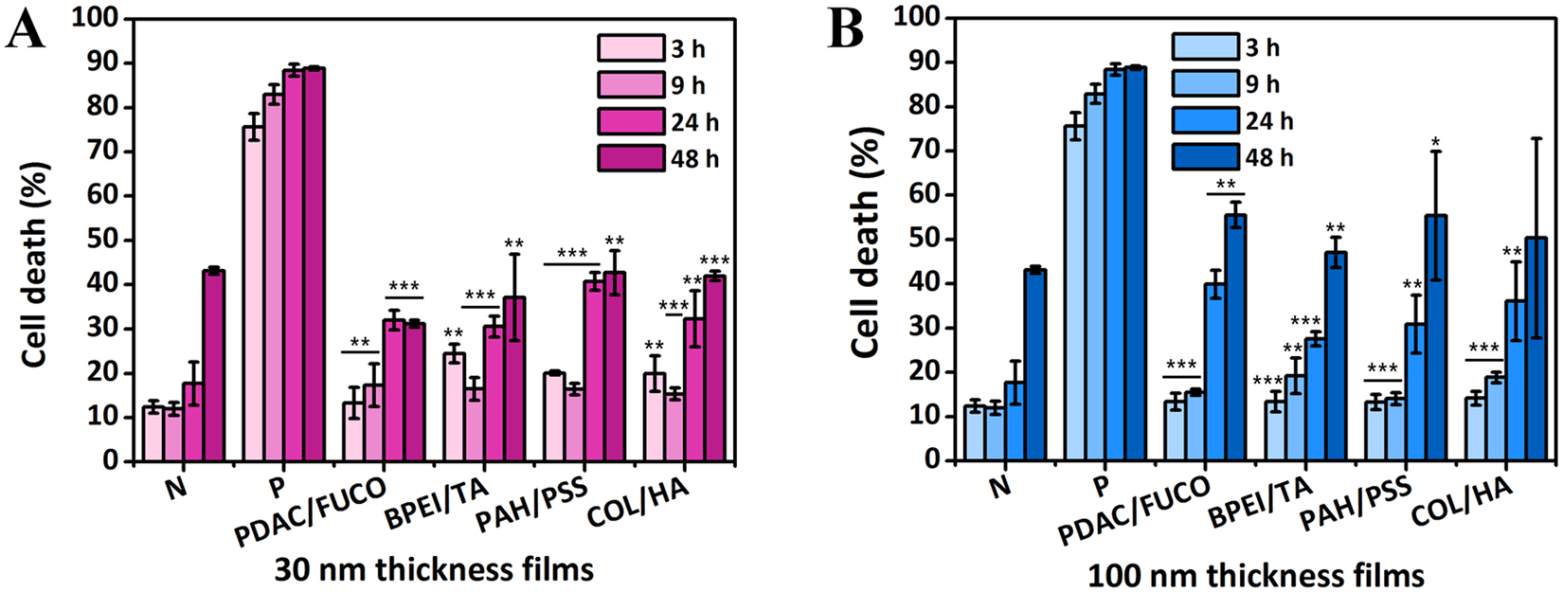
Cytotoxic effects of four types of LbL films with thicknesses of 30 and 100 nm on PBMCs. Cell death was investigated up to 48 h with films with thicknesses of (**A**) 30 nm and (**B**) 100 nm. N and P in each graph indicate the negative control and positive control, respectively (*p < 0.05, **p < 0.01, ***p < 0.001 with respect to 30% MeOH as the positive control; 56.25% of **A** and 75% of **B**, no significant difference from negative control; two-tailed *t*-test).

Contrary to our expectations, there was not much difference in cell death between the toxic and non-toxic film combinations (Fig. 9). For 30-nm-thick films, cell death levels were similar to those of the negative control, except at 24 h, as shown in Fig. 9A. At 24 h, the films seemed to show toxic effects, but after 48 h, no severe cytotoxic effect was observed compared to the control. Figure 9B shows the cytotoxic effects of 100-nm-thick films, and there were no differences among the types of films. Over 24 h, the 100-nm-thick films showed similar cytotoxic tendencies to the 30-nm-thick films shown in Fig. 9A. All types of films induced more cell death than that of the negative control at 24 h, but by 48 h, similar levels of cell death were found for all films and the negative control. Based on this, we concluded that the films were not very harmful to cells overall but that they could accelerate cell death up to 24 h. The reason for this effect could be that cells are affected by direct contact with the film surface both physically and chemically. In order to determine why thicker films had slightly higher cytotoxicity, we investigated the disintegration rates of the films to find the effects of the film surface as well as individual polymers released from the films. The disintegration rates of the films exhibited similar tendencies regardless of thickness (Figure S5). The combination PDAC/FUCO films swelled to 164.7% and 160.2% of the original 30-nm and 100-nm thicknesses, respectively, due to the water retention property of fucoidan (FUCO) derived from *Fucus vesiculosus*, a kind of alga. The BPEI/TA film thicknesses decreased by a narrow range, and the PAH/PSS films based on only synthetic polymers maintained their structures with little swelling. With the increase of water retention, the intermolecular interaction of multilayer films declined, and polymers were likely to be disassembled from the film. However, in the case of Col/HA films, films of both thicknesses were completely reduced within a few hours. Even though collagen and hyaluronic acid are biomaterials, cell death at variable levels can be caused by an increase in the polymer concentration in the media. Fortunately, cell death was not significantly higher because detachments from the film are not harmful to cells forming sub-micrometer size particles^26, 27^.

In summary, cytotoxicity was not affected by the outermost layer of films but presented in a manner dependent on the thickness and disintegration profile. Considering our hypothesis and results, we concluded that cell-surface interaction is likely a near-field interaction in which cells can be affected by polymers that are tens of nanometers into the film surface, while the polymers released from the films could instigate cell death. Of note, we determined that when polymers are assembled in films, toxicity is lower than that of the polymer itself due to the stable state of films and offset of electrical charges.

## Conclusion

In this report, we investigated the cytotoxic effects of twenty types of polymers with different electric charges on blood and immunological cells. Most of the polymers did not show severe toxicity, but several polycations, possibly due to their cell membrane penetration properties, showed stronger toxic effects depending on dose and exposure time. Based on the molecular simulations of polymers interacting with lipid membranes, cytotoxic effects increase in the order of polycation, polyanion, and neutral polymers. Polymer combinations exerted less toxicity, due to counterbalanced charges and an aggregated structure. According to the cell death state assay, all polymers damaged cell membranes but did not induce necrotic cell death. Furthermore, we found that the nanofilms induced low levels of cell death because only a small amount of polymer was incorporated into the film and the films did not break down in a physiological environment. This study of polymer effects on the early immune system provides safety guidelines for *in vivo* applications of biomedical devices produced by LbL, as well as other techniques.

## Materials and Methods

### Materials

Poly(diallyl-dimethyl-ammonium chloride) low-molecular-weight solution (PDAC, Mw 100,000–200,000, 20% (w/w) in H_2_O), branched poly(ethylene imine) (BPEI, Mw ~ 25,000), poly-l-lysine solution (PLL, Mw 70,000–150,000, 0.01 wt% in H_2_O), poly(allylamine hydrochloride) (PAH, Mw ~ 15,000), chitosan low molecular weight (CHI), tannic acid (TA, Mw 1,700), poly(4-styrenesulfonic acid) solution (PSS, Mw ~ 75,000, 18 wt% in H_2_O), dextran from *Leuconostoc spp*. (DEX, Mw ~ 2,000,000), hyaluronic acid sodium salt from *Streptococcus equi* (HA), poly(acrylic acid) (PAA, Mw 1,800), heparin sodium salt from porcine intestinal mucosa (HEP), poly(ethylene glycol) (PEG, Mw 200), and poly(vinyl alcohol) (PVA, Mw 89,000–98,000) were purchased from Sigma-Aldrich (St. Louis, MO). Linear poly(ethylene imine) (LPEI, Mw 250,000) and polyacrylamide (PAAM, Mw ~ 5,000,000, 1% (w/w) in H_2_O) were obtained from Polysciences Inc. (Warrington, PA). COO-functionalized graphene oxide (GO−) and NH_3_+-functionalized graphene oxide (GO+) were synthesized as previously described^28^. Collagen I from rat tail (COL) was supplied by BD Biosciences (San Jose, CA). Poly β-aminoester (PBAE, Mw ~ 10,000) was synthesized as previously described^29^. Fucoidan from *Fucus vesiculosus* (FUCO, Mw 20,000) was supplied by SantaCruz Biotechnology (Santa Cruz, CA). The physicochemical characteristics of the polymers are provided in Table 1.

### Hemolysis test

Red blood cells used in this study were isolated from whole blood by Ficoll–Hypaque (Sigma-Aldrich) gradient centrifugation. Random study samples were chosen from a healthy donor cohort at the Massachusetts General Hospital in Boston, Massachusetts. The Partners Healthcare Institutional Review Board and the Massachusetts Institute of Technology Committee on the Use of Humans as Experimental Subjects approved the study, and each subject gave written informed consent. All methods were performed in accordance with the relevant guidelines and regulations by the Committee on the Use of Humans as Experimental Subjects (COUHES) at MIT.

The hemoglobin concentration was measured using the cyanmethemoglobin (CMH, Drabkin’s reagent, Sigma-Aldrich) method based on a hemoglobin concentration standard (Stanbio Laboratory, Boerne, TX) curve at an absorbance wavelength of 540 nm. The blood was then diluted to a hemoglobin concentration of 10 mg/mL with Ca^2+^/Mg^2+^-free DPBS (Dulbecco’s phosphate-buffered saline; Gibco, Gaithersburg, MD). The polymer and combination solutions at five different concentrations from 0.005 to 50 μg/mL were analyzed using blood from a different donor on each test day. Diluted blood and polymer solutions were added to each well of a 96-well plate. The 96-well plates were incubated in a 37 °C water bath for 210 min. Following the incubation, the supernatants were collected and transferred to a new plate. The supernatants were mixed in a 1:1 ratio with CMH reagent and analyzed with a plate reader (Synergy H1; BioTek, Winooski, VT, USA) at 540 nm. Sample absorbance was corrected for background interference (i.e., films in DPBS without blood). The concentration of cell-free hemoglobin in each sample was determined using the hemoglobin standard curve. Finally, the percent hemolysis was obtained by dividing each sample’s cell-free hemoglobin concentration by the total hemoglobin concentration (10 mg/mL).

### Cell viability test

To assess cell viability against polymers, uncharacterized PBMCs (CTL-UP1; Cellular Technology Ltd., Shaker Heights, OH, USA) were seeded in 12-well culture plates at a density of 1 × 10^4^ cells per well. After 3 hours, the cells were then treated with polymers diluted with cell culture medium at different concentrations (0.01–100 μg/mL). At appropriate time points after exposure to the polymers, the cells were harvested and resuspended in PBS (Gibco), pH 7.4, and then stained with 0.4% trypan blue (Sigma-Aldrich) for 5 min. The percentage of viable cells was determined using a cell counter (JuLI Br, NanoEntek, Seoul, Korea). Cell viability (%) was calculated as follows:

1 $${\rm{Cell}}\,{\rm{viability}}\,( \% )=\frac{{\rm{Live}}\,{\rm{cells}}}{{\rm{Total}}\,{\rm{cells}}}\times 100$$

### AnnexinV-FITC/PI double staining

PBMCs were cultured in 12-well cell culture plates and incubated under 5% CO_2_ at 37 °C for 24 h. The polymers and combinations were diluted to the desired concentrations with cell culture medium and added to each well. Cell culture was continued for 48 h at 37 °C and 5% CO_2_. Once the cells reached at a density of 1 × 10^5^ cells per well, they were collected, centrifuged at 400 × *g* for 5 min, and resuspended in binding buffer. AnnexinV-FITC (BioVision, Milpitas, CA) was added and mixed. After the addition of PI (BioVision) staining solution, the cells were incubated for 15 min in the dark at room temperature (25 °C); binding buffer was then added, and the cells were analyzed using an automated inverted fluorescence microscope (Zeiss Observer Z-1, Carl Zeiss Inc., Novi, MI) to detect cell apoptosis.

### Film preparation

The multilayer films were fabricated onto a silicon wafer using four toxic polymers (PDAC, FUCO, BPEI, and TA) and four non-toxic polymers (PAH, PSS, HA, and COL). The substrate was cleaned and modified to have a negative charge with RCA solution (H_2_O:H_2_O_2_:NH_3_ = 5:1:1% (v/v)) for 10 min at 75 °C. The cleaned substrate was dipped into the polymer solution for 10 min, followed by three rinsing steps for 2, 1, and 1 min. Then, the substrate was dipped into an oppositely charged solution, and the rinsing steps were repeated. Each polymer solution was prepared in distilled water at 1 mg/mL. Sodium chloride was added to the PDAC and FUCO solutions at 1 M and to the PAH, PSS, and HA solutions at 0.5 M. The pH was adjusted using 1 M HCl or 1 M NaOH to reach 7.0 for BPEI, 7.0 for TA, and 4.0 for COL. The multilayer films were dried under a gentle stream of nitrogen, and the thicknesses were measured with a profilometer (Dektak 150; Veeco, Oyster Bay, NY). Each film was prepared to thicknesses of 30 and 100 nm in duplicate.

### Film disintegration

The films described above were dipped into PBMC cell culture medium and incubated (MI-20A; BioBiz, Incheon, Korea) at 37 °C for 48 h. The thickness of each film was measured with a profilometer at certain time intervals.

### Cell viability test for LbL films

To provide direct contact between the cells and LbL films, PBMCs were seeded in 12-well culture plates at a density of 1 × 10^4^ cells per well along with each LbL film for 24 hours. At appropriate time points after polymer exposure, the cells were harvested and resuspended in PBS, pH 7.4, and then stained with 0.4% trypan blue for 3 min. The percentage of viable cells was determined using a cell counter (JuLI Br). Cell death (%) was calculated as follows:

2 $${\rm{Cell}}\,{\rm{death}}\,( \% )=\frac{{\rm{dead}}\,{\rm{cells}}}{{\rm{Total}}\,{\rm{cells}}}\times 100$$

Significant differences in cell death (%) between the controls and each film at equal culture times were evaluated by two-tailed t-tests using Excel.

### Simulation for PEI and PLL

All simulations and analyses were performed with the GROMACS4.6.7 simulation package^30–32^. Models for DMPC lipids and PLL molecules were taken directly from the “MARTINI” coarse-grained (CG) force field (FF)^33–35^, which lumps a few (three or four) heavy atoms into each CG bead. To generate polymer chains that contain different charge states while still maintaining a random-coil structure, cationic beads of the PLL side chain (the CG bead type “Qd”) were replaced with either anionic (“Qa”) or neutral (“P1”) beads. For linear polyethylenimine (PEI), the original parameters for polyethylene glycol (PEG), which were previously developed within the framework of the MARTINI FF by our group^36, 37^, were modified by replacing the originally assigned PEG beads with the cationic “SQd” beads. This modification yields a significant increase in the radii of gyration (~50%), which favorably compares to recent simulations of PEI at different protonation states^38^. To compare the effects of polymer charge, all simulated polymer chains had the same charge density per molecule (Table 2).

Eight polymer chains were positioned above the equilibrated bilayer with a distance of 5 nm between the polymer and bilayer centers (Fig. 5, top). Polymers were evenly distributed on the bilayer to avoid initial clustering. The final simulated system consisted of 8 polymers, 8192 DMPC lipids (4096 DMPC/leaflet), ~29,0000 water beads (equivalent to 1,160,000), and 1024 counterions (Na+ or Cl−) in a periodic box sized 50 × 50 × 18 nm. A temperature of 310 K and a pressure of 1 bar were maintained by applying a velocity-rescale thermostat^39^ and Parrinello-Rahman barostat^40^ on the NPxyPzT ensemble (semi-isotropic pressure coupling). A real space cutoff of 11 Å was used for the Lennard-Jones (LJ) potential with a smooth shift to 0 between 9 and 11 Å. For the Coulomb potential, a short-range interaction with a cutoff of 11 Å and a long-range interaction with the particle mesh Ewald summation (PME)^41^ were used, as our previous work showed that long-range electrostatics need to be included for CG simulations of polymers in lipid bilayers^42–46^. The LINCS algorithm was used to constrain the bond lengths^47^. Simulations were performed for 400 ns with a time step of 10 fs on computational facilities supported by the National Institute of Supercomputing and Networking/Korea Institute of Science and Technology Information with supercomputing resources including technical support (KSC-2016-C3–36). The last 200-ns trajectories were used for the analyses.

## Electronic supplementary material

supporting information
