# Supplementary material for: In vitro blood cell viability profiling of polymers used in molecular assembly
Source: Sci Rep. 2017 Aug 25;7:9481. doi: 10.1038/s41598-017-10169-5 (PMC5573391; doi:10.1038/s41598-017-10169-5)
Supplement: Supplementary file 1 — supporting information [file 41598_2017_10169_MOESM1_ESM.doc]

**Supporting information**

**In vitro blood cell viability profiling of polymers used in molecular assembly**

Hyejoong Jeong^1^, Jangsun Hwang^2^, Hwankyu Lee^3^, Paula T. Hammond^4^, Jonghoon Choi^2^,*, and Jinkee Hong^1^,*

^1^ School of Chemical Engineering & Materials Science, Chung-Ang University, 84 Heukseok-ro, Dongjak-gu, Seoul 06974, Republic of Korea

^2^ School of Integrative Engineering, Chung-Ang University, 84 Heukseok-ro, Dongjak-gu, Seoul 06974, Republic of Korea

^3^ School of Chemical Engineering, Dankook University, 152 Jukjeon-ro, Suji-gu, Yongin-si, Gyeonggi-do 16889, Republic of Korea

^4^ School of Chemical Engineering, Koch Institute for Integrative Cancer Research, Massachusetts Institute of Technology, Cambridge, MA 02139, USA.

***Corresponding author: jkhong@cau.ac.kr, jonghoonc@gmail.com**

Table S1. Combinations commonly used for LbL assembly

Figure S1. Cytotoxic effects of polymers on red blood cells and PBMCs for 9 and 48 h.

Figure S2. Normalized cytotoxic effects of polymers based on PEG.

Figure S3. Cytotoxic effects of combinations on red blood cells and PBMCs for 9 and 48 h.

Figure S4. Normalized cytotoxic effects of combinations based on PEG.

Figure S5. Disintegration rates of four kinds of LbL films with two thicknesses.

**Supporting information Table S1.**

**Table S1.** Combinations commonly used for LbL assembly.

| Combinations | Surface charge (mV) @pH 7.4 |
| --- | --- |
| PDAC+PSS | 29.89 |
| PAH+PAA | -4.61 |
| GO(+)+HEP | -20.38 |
| PLL+HA | 19.32 |
| Poly1+HEP | -14.49 |
| COL+DEX | -13.92 |
| GO+HA | 4.88 |
| COL+GO(-) | -12.60 |
| PLL+DEX | 20.31 |
| COL+PAA | -16.29 |

*Surface charges were measured by a Zeta PALS (Brookhaven).

**Supporting information Figure S1.**


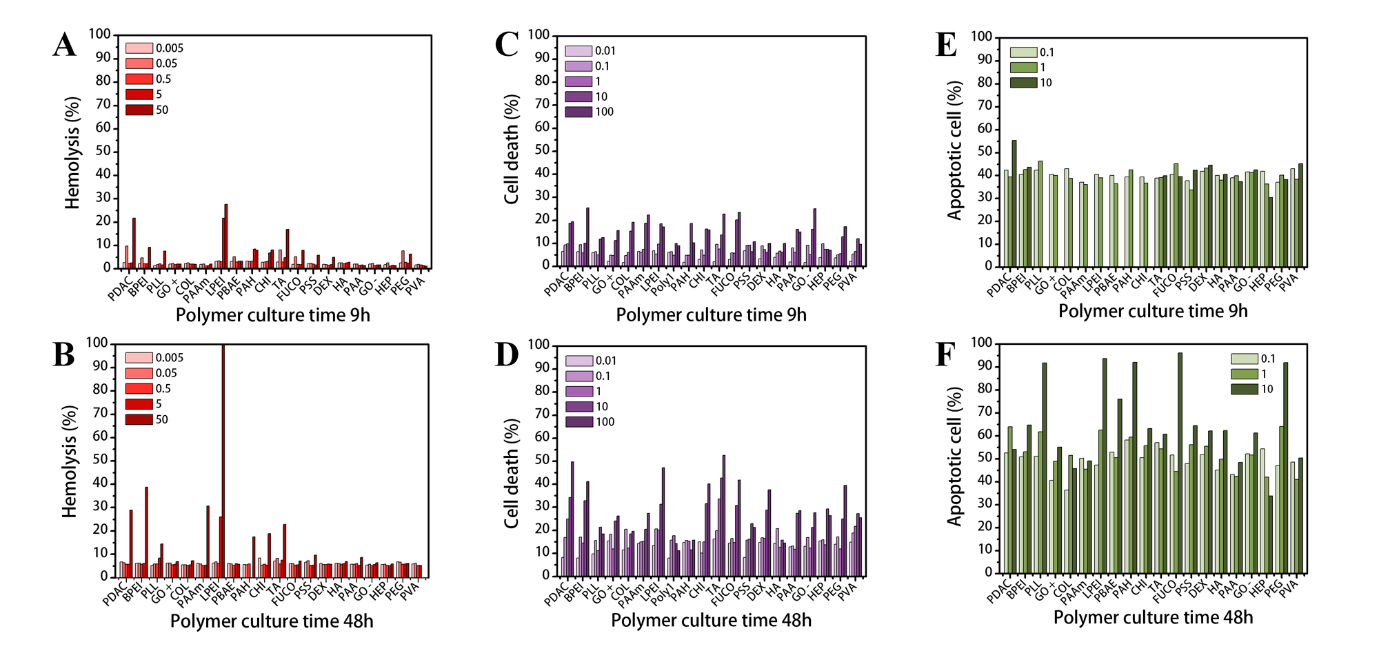


**Figure S1.** Cytotoxic effects of polymers on red blood cells and PBMCs. Hemolysis ratio of different polymer concentrations from 0.005 to 50 ug/mL after (A) 9 h and (B) 48 h. Cell death ratio of five polymer concentrations from 0.01 to 100 ug/mL after (C) 9 h and (D) 48 h. Apoptotic cell ratio of three polymer concentrations from 0.1 to 10 ug/mL after (E) 9h and (F) 48 h. (E) 10 ug/ml solutions of the range from PLL to CHI were omitted due to technical error. Negative control is PEG.

**Supporting information Figure S2.**


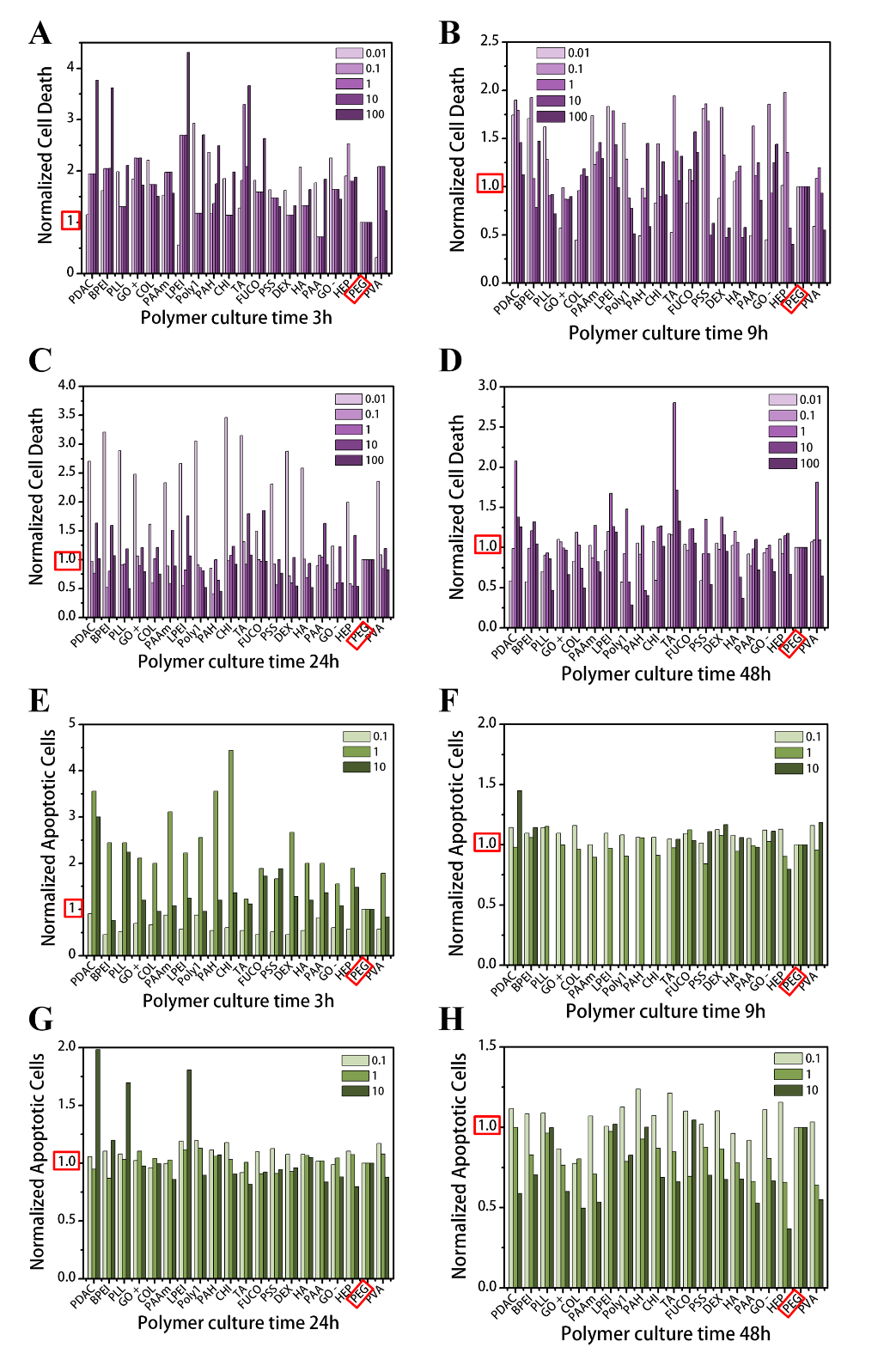


Figure S2. Normalized cytotoxic effects of polymers with regard to cell viability and apoptotic cells comparison with PEG (negative control). Cell viability was presented purple graphs classified culture times; (A) 3 h, (B) 9 h, (C) 24 h, (D) 48 h. Apoptotic cells were presented green graphs classified equal manner; (E) 3 h, (F) 9 h, (G) 24 h, (H) 48 h. The red boxes indicate PEG and in standard cytotoxic level.

**Supporting information Figure S3.**


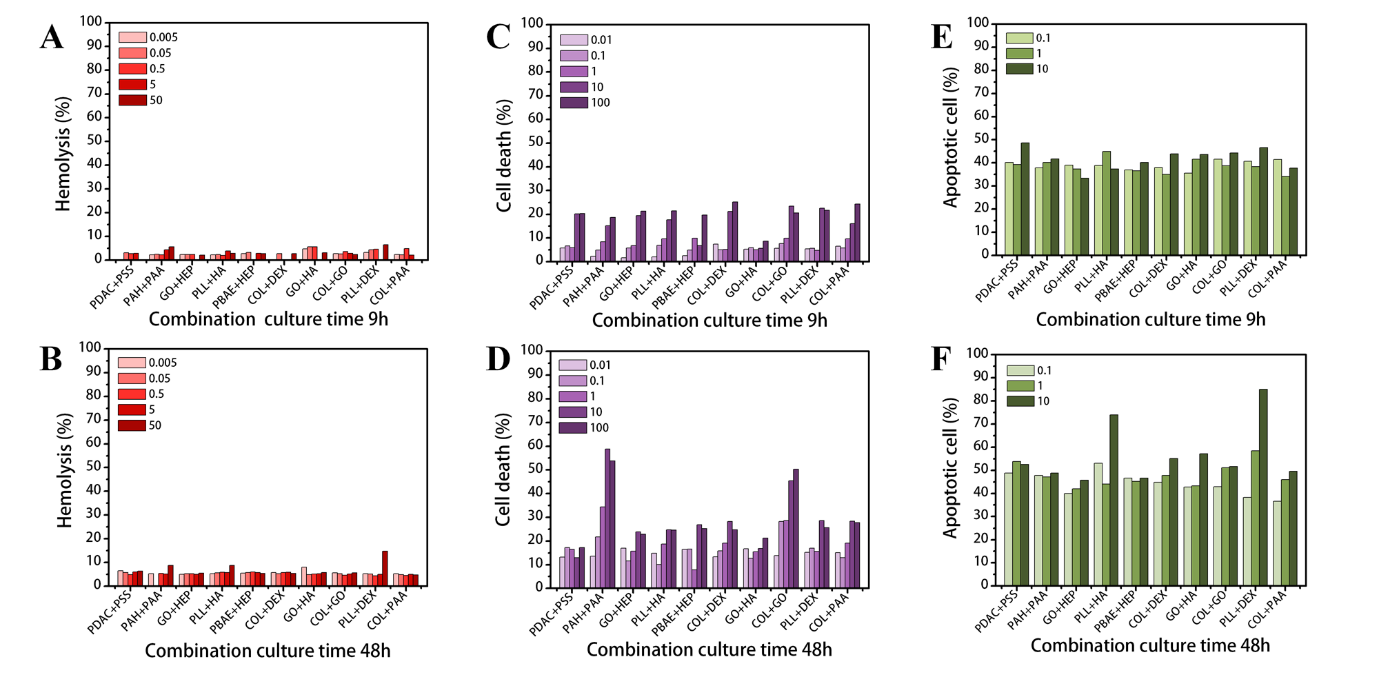


**Figure S3.** Cytotoxic effects of polymer combinations on red blood cells and PBMCs. Hemolysis ratio of different combination concentrations from 0.005 to 50 ug/mL after (A) 9 h and (B) 48 h. Cell death ratio of five combination concentrations from 0.01 to 100 ug/mL after (C) 9 h and (D) 48 h. Apoptotic cell ratio of three combination concentrations from 0.1 to 10 ug/mL after (E) 9 h and (F) 48 h. Negative control is PEG.

**Supporting information Figure S4.**


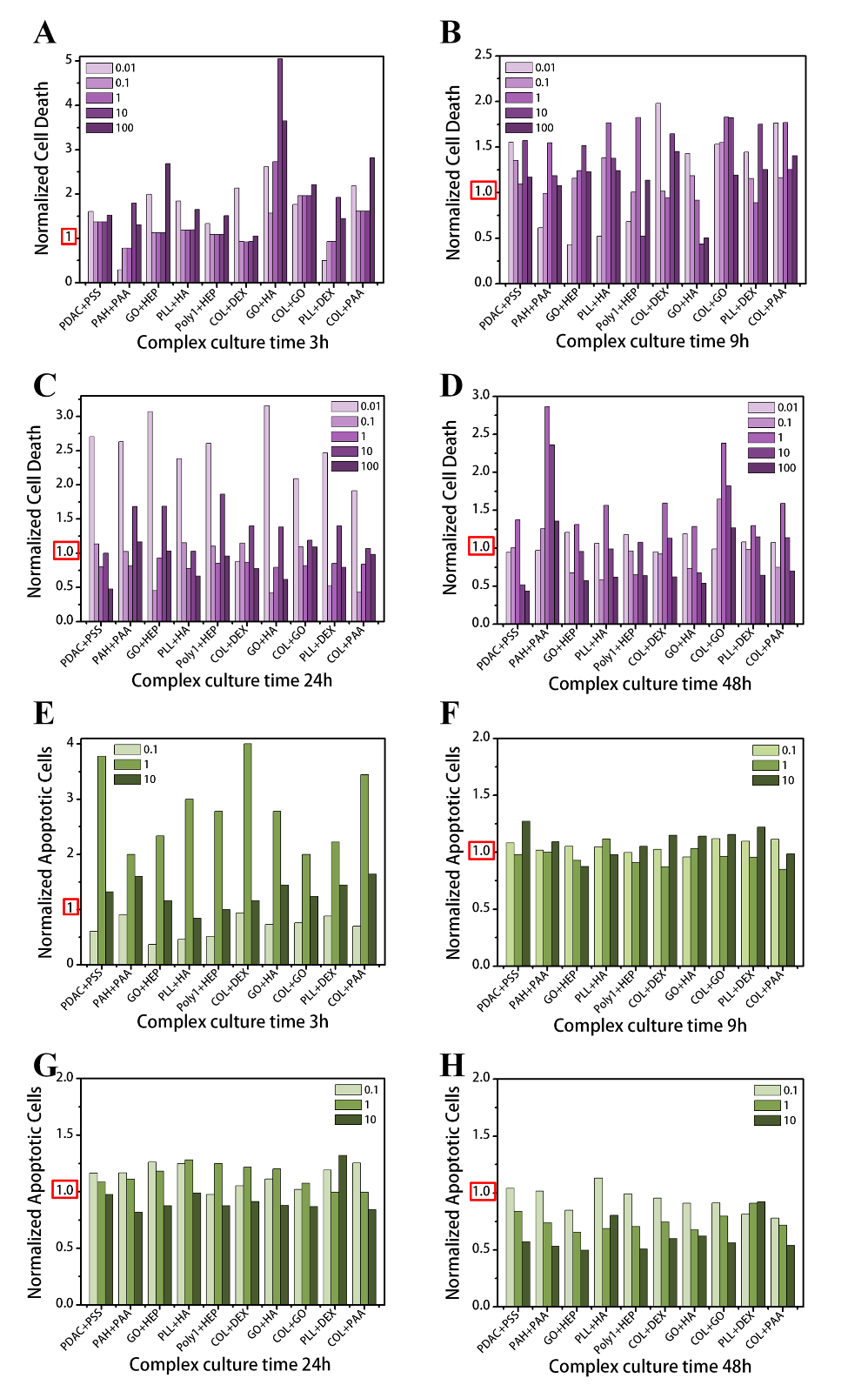


Figure S4. Normalized cytotoxic effects of combinations with regard to cell viability and apoptotic cells comparison with PEG (negative control). Cell viability was presented purple graphs classified culture times; (A) 3 h, (B) 9 h, (C) 24 h, (D) 48 h. Apoptotic cells were presented green graphs classified equal manner; (E) 3 h, (F) 9 h, (G) 24 h, (H) 48 h. The red boxes indicate PEG and in standard cytotoxic level.

**Supporting information Figure S5.**


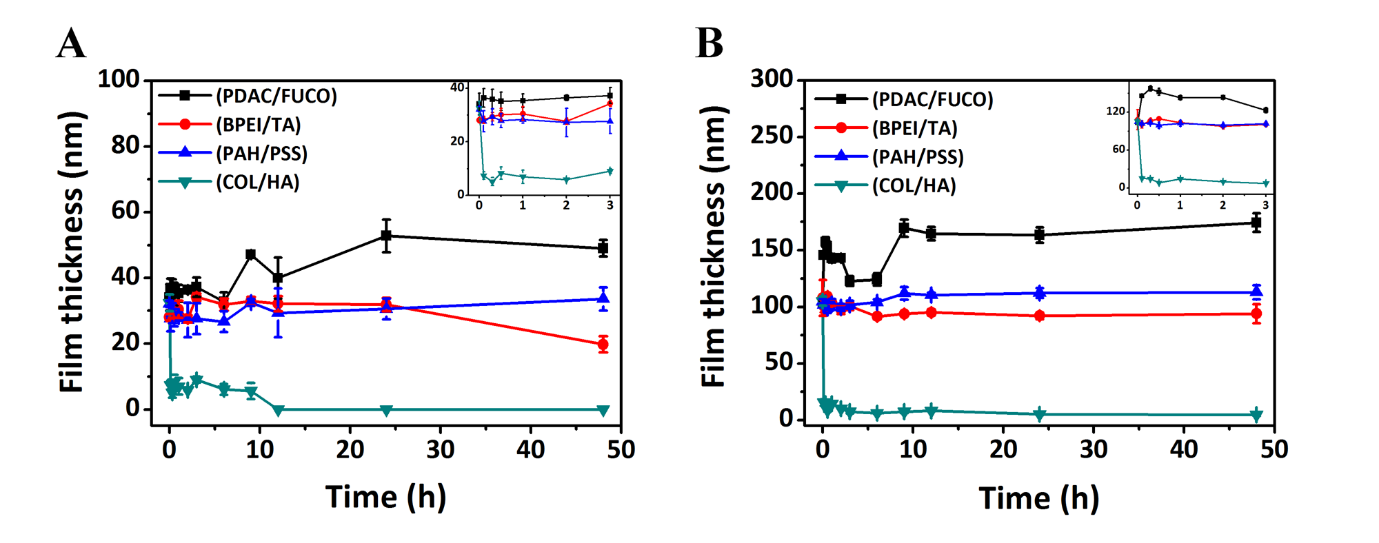


Figure S5. Disintegration rate of LbL films including four kinds of toxic and non-toxic polymer combinations with two thicknesses including (A) 30 nm (B) 100 nm. Insets: initial part of disintegration graph until 3 h, and the axis as equal with outer one. Toxic combinations are (PDAC/FUCO) and (BPEI/TA). Non-toxic combinations are (PAH/PSS) and (COL/HA).
